# *In vivo* probing of purinergic P2X7 as a potential biomarker for suicide risk: a hypothesis

**DOI:** 10.3389/fpsyt.2026.1810859

**Published:** 2026-06-24

**Authors:** Henriëtte D. Heering, Lin Zhang, Dick Swaab, Elsmarieke van de Giessen, Christiaan H. Vinkers, Lot D. de Witte, Sander C.J. Verfaillie

**Affiliations:** 1 Research Department, 113 Suicide Prevention, Amsterdam, Netherlands; 2 Department of Psychiatry, Amsterdam UMC Location AMC, Amsterdam, Netherlands; 3 GGZinGeest Specialized Mental Health Care, Amsterdam, Netherlands; 4 Neuropsychiatric Disorders Lab, Neuroimmunology Group, Netherlands Institute for Neuroscience, an Institute of the Royal Netherlands Academy of Arts and Sciences, Amsterdam, Netherlands; 5 Department of Radiology and Nuclear Medicine, Amsterdam UMC, Location Vrije Universiteit, Amsterdam, Netherlands; 6 Mental Health Program, Amsterdam Public Health, Amsterdam, Netherlands; 7 Mood, Anxiety, Psychosis, Sleep & Stress Program, Amsterdam Neuroscience, Amsterdam, Netherlands; 8 Department of Psychiatry, Amsterdam UMC Location Vrije Universiteit Amsterdam, Amsterdam, Netherlands; 9 Department of Anatomy & Neurosciences,Amsterdam UMC Location Vrije Universiteit Amsterdam, Amsterdam, Netherlands; 10 Radboud University Medical Center, Nijmegen, Netherlands; 11 Icahn School of Medicine at Mount Sinai, New York City, NY, United States; 12 Department of Medical Psychology, Amsterdam UMC Location AMC, Amsterdam, Netherlands; 13 Department of Medical Psychology, University of Amsterdam, Amsterdam UMC, Amsterdam, Netherlands; 14 Brain Imaging, Amsterdam Neuroscience, Amsterdam, Netherlands

**Keywords:** psychological pain, childhood adversity, P2X7, PET imaging, purinergic receptor, suicidality

## Abstract

Suicide claims over 720,000 lives annually worldwide and for every suicide there are many more people who attempt suicide and with suicidal ideation being even more prevalent. To improve the identification of individuals at high risk for suicidal behavior, there is a need to study risk factors in relation to neurobiological mechanisms. This paper proposes an integrative neurobiological hypothesis linking childhood adversity as a known risk factor for suicide attempts and the experience of mental pain in individuals with childhood adversity in their background. The ideation-to-action theory proposes that psychological pain, in addition to hopelessness, is associated with suicide ideation, whereas the acquired capability to attempt suicide enables the progression from suicidal thought to suicidal behavior. A psychological paradox is suggested wherein childhood adversity exposed individuals are more vulnerable for psychological pain, a driver for suicidal ideation, alongside increased physical pain tolerance and fearlessness about death fostering suicide capability. Pain perception may be neurobiologically regulated. Dysregulation of the purinergic pathway and purinergic receptors, such as overactivation of the P2X7 receptor, may contribute to suicidality through at least three parallel mechanisms: neuroinflammation via NOD-, LRR- and pyrin domain-containing protein 3 (NLRP3) inflammasome activation, glutamatergic dysregulation in the anterior cingulate cortex, and disruption of inhibitory pain and fear circuits via brain-derived neurotrophic factor (BDNF) -mediated potassium chloride cotransporter 2 (KCC2) downregulation. Purinergic dysregulation is associated with increased risk for suicidal behavior, which has been demonstrated in bipolar and depressive disorders and schizophrenia. Converging evidence from genetic studies, peripheral inflammatory biomarkers, and postmortem brain tissue indicates that P2X7 dysregulation in suicidality is cell-type and region-specific, with the most direct evidence coming from postmortem findings in individuals who died by suicide independent of psychiatric diagnosis. These neurobiological changes may lower barriers to suicidal behavior during acute crises. *In vivo* PET with [^11^C]SMW139 or [^11^C]JNJ717 can visualize the purinergic pathway through high-affinity binding to the P2X7 receptor. We hypothesize that *in vivo* receptor overactivation is associated with acute suicidality, hence psychological pain. This approach offers potential for biomarker development and targeted therapeutic interventions addressing the neuro-immune substrate of suicidality.

## 1 Introduction

Suicide is a devastating public (mental) health crisis, claiming over 720,000 lives annually and the second leading cause of death for ages 15–29 and the 10th for adults (1). Each suicide affects an estimated 6–10 people and imposes broader societal costs through greater healthcare use, reduced productivity, and increased social services burden (2). Despite this burden, current interventions remain limited. Psychological therapies (e.g., cognitive therapy for suicidality (3; Wiebenga et al., submitted; 4) and pharmacotherapies (lithium for mood disorders; clozapine for schizophrenia) show only partial efficacy (5). This gap underscores the need for novel therapeutic strategies. Biomarker development could help clarify the neuropathophysiological mechanisms underlying suicidality. In the next paragraphs we will discuss the following topics in subsequent order 1) psychological mechanisms and risk factors for suicidality, 2) neurobiological mechanisms of psychological pain, 3) rationale for the involvement of the purinergic pathway in suicidality and 4) specific characteristics of this pathway which can be measured *in vivo* using PET and a potential biomarker.

## 2 From ideation to action; psychological mechanisms and risk factors for suicidality

Suicidal behavior arises from interacting stressors, predisposing factors, and neurobiological vulnerabilities and dysregulation (6–10). However, suicidal ideation is more common than suicide attempts. Cross-national lifetime prevalence is 9.2% for ideation and 2.7% for attempts (11). In major depressive disorder and anxiety disorder, prevalence is 15-17% for ideation and 5-6% for attempts (12, 13). The transition occurs predominantly within the first year after ideation onset (11). Ideation-to-action frameworks, including the Interpersonal Theory (14), the Three-Step Theory (15) and Integrated Motivational-Volitional Model (8), propose psychological mechanisms underlying this transition. The first step toward suicidal ideation is psychological pain, alongside hopelessness, and is defined as lasting, unsustainable feeling from negative self-appraisal (16). According to Klonsky & May (15) psychological pain makes life unbearable and triggers suicidal thoughts, but only when the individual feels hopeless that their pain will improve. Meta-analyses show psychological pain predicts suicidality in adults and adolescents with major depressive disorder (17–19) and distinguish attempters from controls in adolescents (19). These associations persist independent of depression severity (17, 18) and exceed predictive validity of depression or grief alone (20). Ecological momentary assessment confirms psychological pain as independent risk factor beyond depression and hopelessness (21). Following psychological pain, (acquired) suicide capability represents a critical component enabling the transition from ideation to attempt (8, 14, 15, 22). Suicide capability is described as an elevated pain tolerance and diminished fear of death necessary to overcome barriers to lethal self-harm. Capability is acquired rather than innate, developing through habituation to painful stimuli (8, 14, 15, 22). Childhood adversity operates as painful and provocative exposure contributing to suicide capability (23). Repeated fearful and painful experiences induce habituation, reducing pain avoidance and increasing tolerance for lethal self-harm. Attempters show more painful life events than ideators (24) and more frequent non-suicidal self-injury (NSSI) causing perhaps diminishing pain sensitivity (25) supporting suicide capability, e.g. pain habituation, as mechanism enabling the transition from suicidal thought to behavior.

In addition to these psychological mechanisms, demographic and clinical factors differentiate attempters from ideators. Childhood adversity, non-Western descent in Western society, lower education, suicide plan, female gender, earlier ideation onset, and substance use or impulse control disorders independently differentiate attempters from ideators (7, 11, 13, 26). Among these factors, childhood adversity uniquely influences both mechanisms and compass potentially traumatic experiences before the age of 18, including abuse, neglect, exposure to domestic violence, parental substance misuse, mental illness, and separation (27). Physical abuse during childhood predicts attempts beyond ideation (13, 28) through a paradoxical dual effect. Childhood maltreatment induces heightening psychological pain through impaired emotional regulation (29, 30), while simultaneously fostering physical pain habituation through repeated exposure (23, 25).

## 3 Neurobiological mechanisms of psychological pain

Psychological pain engages a neural network partially overlapping with the physical pain network, encompassing anterior cingulate cortex, thalamus, prefrontal cortex, insula, caudate, and putamen (31). This neural overlap indicates that at baseline, individuals more sensitive to one type of pain are also more sensitive to the other (32). MRI studies in adolescents identified two circuits: psychological pain (cerebellum, amygdala and hippocampus) and social disconnection (lateral orbitofrontal cortex and temporal gyri) (33). Comparable abnormalities in the psychological pain circuit abnormalities were observed in adolescents with suicidal ideation, though to a lesser extent than in those with a history of suicide attempts, while social disconnection circuit dysfunction was identified exclusively in adolescent suicide attempters. Lateral orbitofrontal cortex (OFC) involvement has also been demonstrated in another study by showing that greater reduction in OFC activity is associated with higher psychological pain during social exclusion, particularly in suicide attempters (34). Despite correlation, psychological pain and suicidal ideation show partly distinct neural and molecular correlates (35). Psychological pain correlates with activity in prefrontal, anterior cingulate, parietal, and temporal cortices, striatum, and cerebellum, and with expression of serotonergic genes (HTR2B, HTR3A, TPH1) and nociceptin (OPRL1) (35).

## 4 Purinergic receptors and P2X7

Neuroimaging has identified neural substrates of psychological pain, but mechanisms driving aberrant function remain unclear. Ideation-to-action frameworks rely on the stress-diathesis model incorporating environmental stressors alongside psychological mechanisms (8–10, 22). This framework suggests stress-induced neurobiological alterations serve as mediators. Neuro-immune pathways normally protect acute stress but contribute to psychopathology when dysregulated through allostatic overload (36). Hyperactivity of these systems, particularly elevated neuroinflammatory markers, has been associated with depressive and anxiety disorders (37, 38), schizophrenia (39) and bipolar disorder (40). The purinergic pathway may be involved in neuro-immune response, for example through activation of the P2 receptors (e.g. P2X7), located on microglia, triggered by purines (e.g. ATP), and able to drive neuroinflammation by releasing pro-inflammatory cytokines (e.g. interleukine [IL]). Beyond the blood-brain-barrier,in the peripheral blood, downstream elevated IL-6 and C-reactive protein were found to distinguish attempters from non-suicidal individuals (41, 42). The purinergic system, via ATP and adenosine, governs initiation and resolution of neuroinflammatory responses through P1 and P2 receptors on immune cells in the central nervous system, including microglia. There is evidence for the hypothesis that P2X7 receptor dysregulation may be associated to suicidality through at least three parallel mechanisms. First, ATP-driven P2X7 activation may trigger assembly of the NLRP3 inflammasome, resulting in downstream release of IL-1β, IL-6, and TNF-α. This neuroinflammatory cascade has been linked to mood disorders and suicidality and is reflected peripherally in elevated IL-6 and CRP levels found in suicide attempters (41, 42). Second, P2X7 activation may modulate glutamatergic transmission; elevated glutamate in the anterior cingulate cortex has been associated with suicidal ideation in depressed adolescents (43, 44). Third, more speculative, P2X7-driven BDNF release activates TrkB receptors, leading to KCC2 downregulation and GABAergic depolarization (45, 46). Disruption of inhibitory control over nociceptive and fear circuits may contribute to the increased pain tolerance and diminished fear of death that characterize suicide capability. However, this pathway has not yet been tested *in vivo* in suicidal populations, and may offer the neurobiological understanding of how P2X7 contributes to suicide capability. Importantly, these mechanisms are not mutually exclusive and may act simultaneously, which is what makes P2X7 a mechanistically relevant convergence point for suicidality. Specificity may emerge from the convergence of these factors, neurobiological, experiential, and behavioral, rather than from the receptor signal alone. In addition, genetic studies point towards involvement of the P2RX7 gene: Kristof et al. (47) identified associations between P2RX7 gene polymorphisms, affecting the degree of neuroinflammation, and current suicidal ideation. Specific genetic variants (SNPs rs641940, rs1653613, and rs78473339) appeared to mediate the effect of early childhood adversities on later suicide risk (47). It has also been hypothesized that early life stress and childhood adversity may increase the risk of neuropsychiatric disorders via immune activation, whereby excessive ATP release activates P2X7R signaling in the brain, contributing to long-term neuroinflammation and the development of anxiety and depression (48). Neuro-immune pathways may reflect a stress-sensitized brain state that elevates risk for psychiatric conditions and may underpin the previously discussed pain perception paradox observed in individuals with childhood adversity histories. The most direct evidence linking P2X7 to suicidality, rather than to associated psychiatric disorders, comes from postmortem human brain studies. Zhang et al. (49) demonstrated altered P2RX7 expression in the hippocampal dentate gyrus of individuals who died by suicide, independent of diagnosis (MDD or BD). In the dorsolateral prefrontal cortex, this alteration was restricted to MDD, suggesting a partially diagnosis-specific pattern alongside a transdiagnostic pathophysiological signal. This is supplemented by genetically mediated evidence: Kristof et al. (47) showed that P2RX7 polymorphisms specifically moderate the impact of childhood adversity on current suicidal ideation. Furthermore, indirect evidence linking P2X7 dysregulation to suicidality comes from studies on bipolar disorder, schizophrenia, and MDD. While consistent with this picture, this constitutes indirect support, as these conditions are associated with elevated suicide risk but are not equivalent to suicidality per se. It should be noted that elevated P2X7 activity has also been reported in neurological conditions such as Alzheimer’s disease and Parkinson’s disease (46, 50), in which suicidality is not a primary feature. How P2X7 dysregulation in suicidality differs from these conditions remains an open question. Furthermore, P2RX7 has been associated with other psychiatric symptoms including psychotic features (51, 52), suggesting that isoform-specific expression of P2RX7 may account for differential pathogenic effects across conditions.

## 5 *In vivo* quantification of purinergic P2X7 receptor

Evidence linking genetic vulnerability, childhood adversity, neuroinflammation and peripheral neuroinflammatory biomarkers points toward P2X7 receptor dysfunction as an integrative mechanism underlying the transition from suicidal ideation to action. However, a critical gap remains while we have genetic associations and peripheral inflammatory markers, the actual P2X7 receptor dysfunction in the brains of suicidal individuals has never been directly quantified *in vivo* in individuals with suicidality. Specific regional P2X7 activity can however be quantified *in vivo* using PET with [^11^C]SMW139 (53) or using [^11^C]JNJ717 (54), enabling assessment of neuroimmune dynamics in brain regions linked to various neuropsychiatric conditions (5).

“[¹¹C]SMW139 and [¹¹C]JNJ717 bind with high affinity to the P2X7 receptor, which is expressed on pro-inflammatory microglia in various brain regions. Postmortem evidence of P2RX7 dysregulation in relation to suicide comes from Zhang et al. (49), who examined the hippocampal-hypothalamic-prefrontal circuit in individuals who died by suicide or legal euthanasia. P2X7 alterations were found in both the prefrontal cortex and the medial temporal lobe. In the hippocampal dentate gyrus (DG), P2RX7 was elevated in granule cell nuclei of individuals with fatal suicidality, independent of psychiatric diagnosis, but quantification of glial cells revealed no significant differences. In the prefrontal cortex, P2RX7 transcripts were reduced in MDD, particularly in those who died by suicide, which the authors interpret as diminished prefrontal inhibition of subcortical stress circuits. Importantly, suicide-specific molecular features faded with prolonged postmortem obduction delay, suggesting that the actual spatial extent of P2X7 dysregulation in the living brain may be broader than postmortem findings indicate. In addition, differences may generally exist between antemortem and postmortem P2X7 spatial distribution, particularly in cases involving an extreme cause of death such as suicide. Translating these postmortem findings to *in vivo* PET imaging poses specific challenges. Current PET tracers bind to P2X7, and this receptor is highly expressed on microglia, particularly on activated microglia during neuroinflammation. However, P2X7 is also expressed, to a lesser extent, on other cell types such as astrocytes and oligodendrocytes. Furthermore, the DG is a small anatomical region that can be delineated with high-resolution MRI, but remains challenging to capture with PET due to limited spatial resolution and interpretation maybe difficult due to spill-in/spill-out effects. Additional complexity arises from the existence of multiple P2RX7 isoforms, which may differ in expression patterns, function, and ligand binding properties across conditions, and may account for differential pathogenic effects in suicidality compared to other neurological and psychiatric disorders (52). Notwithstanding, some studies have demonstrated that brain processes can be quantified in the DG (55). The entorhinal cortex, which showed suicide-specific microglial pathology in the same postmortem dataset (49) and is anatomically more convenient to quantify, may represent a more suitable region of interest (ROI). Of note, antemortem P2X7 dysregulation likely extends beyond relatively small regions such as the DG and the entorhinal cortex. Whether current P2X7 PET tracers are able to capture P2X7 dysregulation associated with suicidality remains to be established. *in vivo* quantification of P2X7 dysregulation could be an important adjunct to the indirect peripheral markers, including elevated IL-6 and CRP, already observed in suicide attempters.

## 6 The P2X7 hypothesis: unifying psychological pain and suicide capability

**Figure 1:**
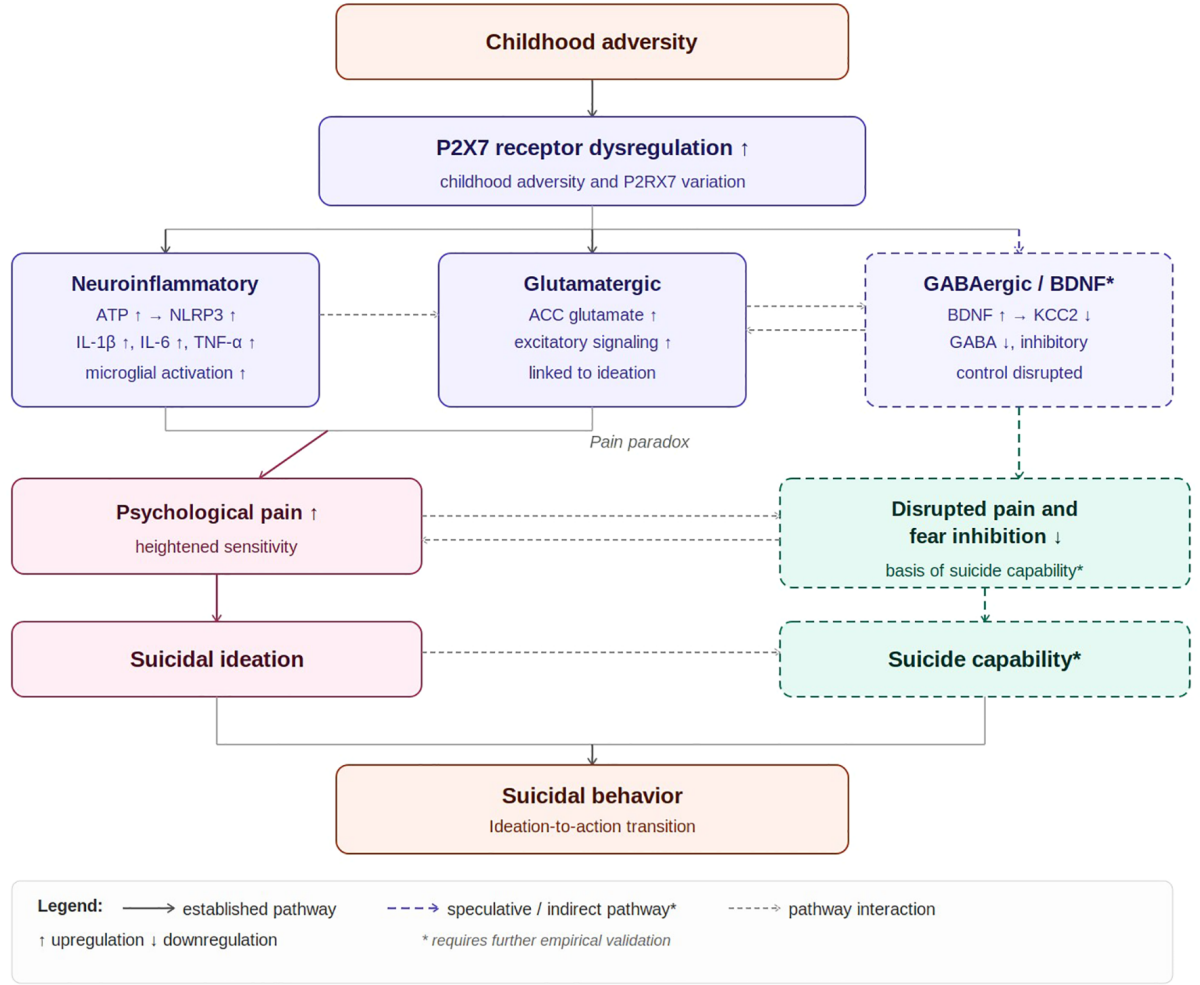
Proposed model linking P2X7 receptor dysregulation to suicidal behavior via three parallel pathways. Childhood adversity and P2RX7 genetic variation may sensitize P2X7 receptor activity, potentially engaging neuroinflammatory (ATP → NLRP3 → IL-1β/IL-6/TNF-α), glutamatergic (ACC excitatory signaling), and GABAergic/BDNF (KCC2 downregulation, disrupted inhibitory control) pathways. The first two pathways may converge on heightened psychological pain sensitivity, potentially driving suicidal ideation. The third may contribute to disrupted pain and fear inhibition, which could form a neurobiological basis for suicide capability. Together, these constitute the proposed pain paradox, converging within the ideation-to-action framework on suicidal behavior. All pathways are hypothetical; dashed lines indicate pathways with a more limited empirical basis that require further validation. Pathway interactions (horizontal dashed arrows) represent suggested cross-pathway effects that have not yet been directly tested. Although childhood adversity is the primary risk factor examined in this hypothesis, psychopathology may similarly sensitize P2X7 receptor activity and contribute to the proposed pathways.

The proposed theory integrates three lines of evidence as is depicted in **Figure 1**: First, childhood adversity contributes to both heightened psychological pain and increased physical pain tolerance, two key drivers of suicidal behavior. Second, neuroimaging studies link psychological pain to neural circuits involved in nociceptive processing. Third, post-mortem findings, genetic studies, and peripheral inflammatory markers implicate P2X7-mediated neuro-immune dysregulation in suicide risk. In sum, P2X7 dysfunction may represent an integrative neurobiological mechanism linking psychological constructs in ideation-to-action frameworks to suicidal behavior. This dysregulation affects pain processing circuits (ACC, PFC, corticolimbic) and inhibitory circuits involved in fear and nociception, and may alter downstream neurochemical neurotransmission, potentially explaining neurobiological alterations observed in suicide attempters (56, 57).

## 7 Research and clinical implications

Investigating the purinergic pathway including P2X7, for example with *in vivo* [^11^C]SMW139 PET, could transform suicide prevention strategies and may offer a novel biological treatment window. Regional P2X7 receptor quantification may provide an objective biomarker for acute suicide risk, differentiating between ideators and attempters. Moreover, P2X7 receptor activity may also be altered by antagonists, such as AK1780, AZD9056, and A-438079 (58–60), designed to treat neuroinflammation, chronic pain, and autoimmune diseases by inhibiting ATP-induced proinflammatory cytokine release. Future studies could be based upon the following empirically testable hypothesis: (1) compared to non-suicidal controls, acutely suicidal patients likely show elevated *in vivo* P2X7 receptor activity, in regions associated with psychological pain; (2) P2X7 activity should correlate with both self-reported psychological pain intensity and objective pain tolerance measures; (3) severity of childhood adversities should mediate the relationship between P2X7 dysfunction and suicide attempt history; and (4) specific regional P2X7 patterns may identify individuals at imminent risk of transitioning from ideation to action. The influence of P2X7-mediated neuroinflammation could be investigated in conjunction with large-scale brain networks underlying emotional regulation and decision-making during the transition from suicidal ideation to action. Lastly, although this hypothesis focuses on P2X7 as a unifying mechanism, suicidality may involve other pathways (HPA axis dysregulation, glutamatergic excitotoxicity, epigenetic modifications) that interact with neuro-immune signaling. The proposed biomarker is primarily a risk biomarker, identifying individuals with elevated neurobiological vulnerability for suicidal behavior based on P2X7 dysregulation in the context of childhood adversity and psychological pain. It may additionally serve as a state biomarker during acute suicidal crises.

An important conceptual question concerns the temporal dynamics of P2X7 alterations in relation to suicidality. P2X7 dysregulation may function as both a trait vulnerability marker, reflecting chronic neuroinflammatory sensitization linked to childhood adversity and genetic background, and a potential state marker during acute suicidal crises. The trait component aligns with the ideation phase, wherein persistent psychological pain is driven by a sensitized neuroimmune baseline. State-level variation, if present, would correspond to the ideation-to-action transition. Future studies should consider longitudinal designs with repeated neuroimaging or crisis-timed protocols to examine state-related changes in P2X7 activity.

Given that P2RX7 polymorphisms and isoforms affect receptor expression, channel function, and ligand binding properties, future PET studies may include genotyping of known variants (including rs641940, rs1653613, rs78473339 as identified by 47) to investigate if these variants affect *in vivo* quantification profiles of P2X7. This will allow proper interpretation of PET signal variability and enable examination of how genetic moderation of P2X7 function interacts with the neuroinflammatory signal in individuals with childhood adversity and suicidality.

## 8 Conclusion

The P2X7 receptor hypothesis offers a neurobiological framework integrating psychological pain, suicide capability, and childhood adversity within ideation-to-action models. By proposing a specific molecular mechanism linking stress, neural circuits, and pain processing, this framework connects psychological constructs to measurable biological substrates. Demonstrating P2X7 dysfunction in acute suicidal states could open therapeutic windows targeting the neuro-immune mechanisms underlying the transition from ideation to action.
